# Fulminant hepatic failure in association with quetiapine: a case report

**DOI:** 10.1186/1752-1947-6-418

**Published:** 2012-12-12

**Authors:** Fawaz Al Mutairi, Girish Dwivedi, Turki Al Ameel

**Affiliations:** 1 University of Ottawa Heart Institute, Ottawa, Ontario, Canada; 2 Division of Gastroenterology, University of Alberta, Edmonton, Alberta, Canada

**Keywords:** Acute liver injury, Fulminant hepatic failure, Quetiapine

## Abstract

**Introduction:**

Fulminant hepatic failure is a serious disease with significant mortality and morbidity. Identifying the exact cause of hepatic failure and predicting prognosis is of paramount importance in managing such patients. Drug-induced liver injury is a common but challenging entity to treat. The use of newer drugs and medications with previously unknown hepatotoxicity add to the challenges faced by treating physicians. Quetiapine is an antipsychotic that has rarely been linked to acute liver injury. In the present work we describe a case of fulminant hepatic failure secondary to use of quetiapine.

**Case presentation:**

A 59-year-old Caucasian woman with known Parkinson’s disease was being treated with quetiapine for hallucinations. She was referred to our hospital with yellow discoloration of the sclera and later on developed clinical features suggestive of hepatic encephalopathy. A diagnosis of fulminant hepatic failure was made following her admission to the intensive care unit. Her condition improved after discontinuing the drug and providing the standard supportive treatment.

**Conclusions:**

Our findings in the present report emphasize the importance of keeping an open mind in cases of fulminant hepatic failure. As drug-induced hepatotoxicity is the most common cause of fulminant hepatic failure in many parts of the world, consideration should be given to the medication(s) patients receive as the potential cause and a review of this list should be part of the clinical care given.

## Introduction

Fulminant hepatic failure (FHF) is defined by the rapid development of severe acute liver injury accompanied by synthetic dysfunction and the development of encephalopathy in an individual with either a previously normal liver or well compensated liver disease [1,2]. Proposed definitions of the time course for fulminant hepatic failure include the development of encephalopathy within eight weeks of the onset of symptoms in a patient with a previously healthy liver [1] or the appearance of encephalopathy within two weeks of developing jaundice, in a patient with or without previous underlying liver dysfunction [2]. Although, FHF can result from a wide variety of causes, drug-induced hepatoxicity is common, causing high mortality with an estimated 3.5 deaths per million people in the US alone [3]. The reported short-term survival is 49% although the outcome of FHF has significantly improved with the introduction of liver transplantation [4].

Quetiapine (Seroquel®; AstraZeneca) is an atypical antipsychotic agent used to control positive and negative symptoms in patients with psychosis [5]. It has a low side effect profile and is generally well tolerated in doses from 150 to 750mg/day [6]. The common known side effects of quetiapine include a mild asymptomatic disturbance of liver enzymes, leukopenia, pancytopenia and thrombotic thrombocytopenic purpura [7,8]. Rarely, neuroleptic malignant syndrome, hyperprolactinemia, myoclonus and cardiac arrhythmias have also been reported as adverse effects [9-12]. We describe the case of a patient with FHF caused by quetiapine, a rarely reported adverse effect of this drug associated with significant mortality if not identified and treated early [13-15].

## Case presentation

A 59-year-old Caucasian woman with a prior history of Parkinson’s disease was on carbidopa-levodopa combination for three years and oxazepam along with pramipexole for six months before she developed hallucinations that were attributed to pramipexole therapy. Subsequently, pramipexole was discontinued and our patient was given quetiapine to treat her hallucinations. She had received six weeks of quetiapine therapy before presenting to our hospital with the main complaint of feeling unwell for three weeks. Her symptoms were accompanied with nausea, vomiting, decrease appetite and abdominal pain for a few days duration. She had also noticed a yellowish discoloration of her sclera but no change in the color of urine or stool. There was no accompanying history of fever, chills or rigors. No risk factors or history suggestive of familial liver disease was provided. She was a non-smoker and denied drinking alcohol.

The results of a general physical examination revealed a stable hemodynamic status with bilateral icterus but no pallor, cyanosis or lymphadenopathy. An abdominal examination demonstrated distention with tenderness in the right upper quadrant but no clinically detectable ascites or hepatosplenomegaly. No other stigmata of chronic liver disease were seen. Cardiovascular and respiratory system examinations were unremarkable. No abnormality was detected on the neurological examination.

Admission blood test results showed a normal hemogram and normal renal function tests including electrolytes but deranged liver function tests (alanine aminotransferase: 71IU/L; aspartate aminotransferase: 740IU/L; lactate dehydrogenase: 737IU/L; γ-glutamyl transferase: 509IU/L; alkaline phosphatase: 196IU/L; total bilirubin: 244μmol/L and an international normalized ratio (INR) of 2.7). Her blood ethanol level was in the normal range. Viral serology test results for hepatitis A, B and C were negative. A vasculitic screen was performed, which revealed an anti-nuclear antibody titer of 1:1600 and positive anti-double-stranded deoxyribonucleic acid (dsDNA) results, but negative results for anti-liver kidney microsomal antibody. Tests for anti-neutrophil cytoplasmic antibodies showed negative results for anti-myeloperoxidase but were equivocal for anti-proteinase 3. Normal α-1 anti-trypsin levels (1.89μmol/L) and normal levels for other immunoglobulins were found (IgG: 34g/L, IgA: 2.44g/L and IgM: 1.5g/L). The results of screening tests for Wilson’s disease and hemochromatosis were also negative.

An ultrasound scan of the abdomen showed a distended gall bladder with absence of stones and no free fluid in the abdomen. The liver and spleen sizes were reported as within normal ranges. Computerized tomography of the abdomen confirmed the absence of intra-hepatic biliary dilatation.

In view of the above-mentioned findings and her history, our patient was admitted with a probable diagnosis of quetiapine-induced hepatitis. Quetiapine was discontinued but other medications, that is, carbidopa-levodopa and oxazepam, were not stopped. The initial treatment was mainly supportive. However, within 48 hours of admission, our patient demonstrated signs of encephalopathy in the form of confusion, astrexis and impaired level of consciousness with the development of significant ascitis. She was subsequently transferred to the intensive care unit and the local liver transplant team was consulted for probable transplant. After ruling out spontaneous bacterial peritonitis, our patient was treated with lactulose, spironolactone and furosemide. She was also given methyprednisolone 20mg intravenously. A follow-up abdominal ultrasound scan revealed splenomegaly, coarse liver parenchyma and ascitis but no focal lesions or cholelithiasis. The hepatic and portal circulations were patent on Doppler examination. A trans-jugular liver biopsy confirmed extensive confluent and bridging necrosis predominantly in zone 3. The portal area contained mild to focally moderate mixed inflammatory cellular infiltrate consisting of lymphocytes, macrophages, eosinophils and a few polymorphonuclear cells along the boundaries of the necrotic areas. The parenchyma showed variable degrees of hepatocellular degeneration and mild mixed inflammatory cellular infiltrate consisting mainly of lymphocyes and macrophages with few plasma cells and prominent Kupffer cells. The overall histopathological picture was reported to be suggestive of an acute hepatitis with confluent and bridging necrosis, indicative of drug-induced liver injury.

Our patient’s clinical symptoms improved gradually accompanied by improvement in biochemical parameters over the course of her stay in the intensive care unit. The encephalopathy resolved completely with improvement in icterus, nutritional status and oral intake. Later on, she was transferred out of the intensive care unit to the general ward and put on a tapering dose of oral prednisolone starting at 40mg/day.

She was discharged home in a stable condition approximately six weeks after the initial admission on 20mg prednisolone per day with a suggested tapering of 5mg every week. She was advised against taking quetiapine in the future. Follow-up blood test results eight weeks after discharge revealed mildly elevated liver enzymes (less than twice the upper limit of normal) with normal synthetic and excretory hepatic functions.

## Discussion

Quetiapine is a dibenzothiazepine, an atypical antipsychotic agent that is known to have a positive effect on positive and negative symptoms of psychosis [5]. Although its mechanism of action is poorly understood, it is thought to exert its effect through a combination of dopamine (D2) and 5-hydroxytryptamine-2 (5HT2) receptor antagonism. Quetaipine demonstrates a heterogenic antagonist activity in the brain with stronger antagonism of 5HT2 receptors compared to D2. It also has affinities for a range of other neurotransmitter receptors such as serotonin 5HT1a, D1, histamine-1 and adrenergic α-1 and α-2. It has, however, no appreciable affinities for cholinergic muscarinic and benzodiazepine receptors [16]. It is metabolized via the hepatic mixed function oxidase system using the cytochrome P450 3A4 isoenzyme [5]. Quetiapine [17] was considered to be a non-hepatotoxic drug except for a mild and transient asymptomatic elevation of liver enzymes prior to the first published report of quetiapine-induced FHF by El Hajj *et al*. in 2004 [13].

The most likely etiology in our patient’s case was quetiapine-induced liver injury. Other possible etiologies such as viral hepatitis A, B and C, metabolic disorders including Wilson’s disease and vascular diseases such as Budd-Chiari syndrome were ruled out. The other diagnosis considered with such a presentation is ‘FHF of unknown etiology’, which accounts for 17% of FHF cases [18]. However, the temporal association between the commencement of the medication and the onset of her symptoms strongly indicates the possibility of quetiapine as the culprit agent. The other differential diagnosis that was considered was of autoimmune hepatitis due to the elevated anti-nuclear antibody level found in our patient. However, anti-nuclear antibody elevation is a non-specific finding and can also be seen in drug-induced hepatic injury. Moreover, in absence of other serological markers and absence of findings on liver biopsy, an autoimmune etiology is very unlikely. Of note, acute liver injury secondary to autoimmune process or drug toxicity shows response to steroid therapy in both cases. Also, extensive zone 3 predominant necrosis (as seen in our patient) on liver biopsy is seen in drug-induced liver injury [19]. Indeed, on the Roussel Ucalf Causality Assessment Method/Council for International Organizations of Medical Sciences scoring system our patient had a score of 7, indicating probable causation of FHF by quetiapine [20,21].

In cases of FHF, the decision for liver transplantation is based on clinical judgment and utilization of prognostic criteria such as the King’s College criteria. For known acetaminophen-induced FHF, the criteria include: disease etiology (cryptogenic/toxin), age (<10 years, >40 years), duration of jaundice (more than one week before the development of encephalopathy), serum bilirubin concentration of 18mg/dl and INR >3.5, our patient fulfilled only three of these five criteria [22]. Complete resolution except for minimal elevation of liver enzymes was the final outcome in our patient as opposed to mortality in two other cases of quetiapine-induced liver injury reported in the literature [13]. One other case reported by Shpaner *et al*. showed improvement after quetiapine cessation, which may have been due to subfulminant rather than fulminant hepatic failure [15].

## Conclusions

Our patient’s case emphasizes the importance of keeping an open mind in cases of fulminant hepatic failure. As drug-induced hepatotoxicity is the most common cause of fulminant hepatic failure in many parts of the world, consideration should be given to the medication(s) patients receive as being the potential cause, and a review of the list should be part of the clinical care administered.

## Consent

Written informed consent was obtained from the patient for publication of this case report and any accompanying images. A copy of the written consent is available for review by the Editor-in-Chief of this journal.

## Competing interests

The authors declare that they have no competing interests.

## Authors’ contributions

FA: Designed the study, reviewed the literature and drafted the first manuscript. GD: Reviewed and edited the manuscript. TA: Reviewed the literature, reviewed and edited the manuscript. All authors read and approved the final manuscript.

## References

[B1] Trey C Davidson LS Popper H, Shaffiner F The management of fulminant hepatic failure Prognosis in Liver Disease 1970 Grune and Stratton, New York, NY 282 4908702

[B2] Bernauau J Reuff B Benhamou JP Fulminant and subfulminant liver failure: definitions and causes Semin Liver Dis 1986 6 97 10.1055/s-2008-1040593 3529410

[B3] Hoofnagle JH Carithers RL Jr Shapiro C Ascher N Fulminant hepatic failure: summary of a workshop Hepatology 1995 21 240 252 7806160

[B4] Schiodt FV Atillasoy E Shakil AO Schiff ER Caldwell C Kowdley KV Stribling R Crippin JS Flamm S Somberg KA Rosen H McCashland TM Hay JE Lee WM Etiology and outcome for 295 patients with acute liver failure in the United States Liver Transpl Surg 1999 5 29 34 10.1002/lt.500050102 9873089

[B5] Lacy C Armstrong L Goldman M Lance L Quetiapine Drug Information Handbook 2001 9 Lexi-comp Inc, Hudson, OH 1046 1047

[B6] Mullen J Jibson MD Sweitzer D A comparison of the relative safety, efficacy, and tolerability of quetiapine and risperidone in outpatients with schizophrenia and other psychotic disorders: the quetiapine experience with safety and tolerability (QUEST) study Clin Ther 2001 23 1839 1854 10.1016/S0149-2918(00)89080-3 11768836

[B7] Iraqi A A case report of pancytopenia with quetiapine use Am J Geriatr Psychiatry 2003 11 694 14609812 10.1176/appi.ajgp.11.6.694

[B8] Huynh M Chee K Lau DH Thrombotic thrombocytopenic purpura associated with quetiapine Ann Pharmacother 2005 39 1346 1348 10.1345/aph.1G067 15914516

[B9] Bourgeois JA Babine S Meyerovich M Doyle J A case of neuroleptic malignant syndrome with quetiapine J Neuropsychiatry Clin Neurosci 2002 14 87 10.1176/appi.neuropsych.14.1.87 11884665

[B10] Alexiadis M Whitehorn D Woodley H Kopala L Prolactin elevation with quetiapine Am J Psychiatry 2002 159 1608 1609 10.1176/appi.ajp.159.9.1608 12202292

[B11] Velayudhan L Kirchner V Quetiapine-induced myoclonus Int Clin Psychopharmacol 2005 20 119 120 10.1097/00004850-200503000-00011 15729090

[B12] Gajwani P Pozuelo L Tesar GE QT interval prolongation associated with quetiapine (Seroquel) overdose Psychosomatics 2000 41 63 65 10.1016/S0033-3182(00)71175-3 10665270

[B13] El Hajj I Sharara AI Rockey DC Subfulminant liver failure associated with quetiapine Eur J Gastroenterol Hepatol 2004 16 1415 1418 10.1097/00042737-200412000-00029 15618854

[B14] Naharci MI Karadurmus N Demir O Bozoglu E Ak M Doruk H Fatal hepatotoxicity in an elderly patient receiving low-dose quetiapine Am J Psychiatry 2011 168 212 213 10.1176/appi.ajp.2010.10091292 21297052

[B15] Shpaner A Li W Ankoma-Sey V Botero RC Drug-induced liver injury: hepatotoxicity of quetiapine revisited Eur J Gastroenterol Hepatol 2008 20 1106 1109 10.1097/MEG.0b013e3282f8e3a0 19047843

[B16] McConville BJ Arvanitis LA Thyrum PT Yeh C Wilkinson LA Chaney RO Foster KD Sorter MT Friedman LM Brown KL Heubi JE Pharmacokinetics, tolerability, and clinical effectiveness of quetiapine fumarate: an open-label trial in adolescents with psychotic disorders J Clin Psychiatry 2000 61 252 260 10.4088/JCP.v61n0403 10830145

[B17] Zeneca Pharma Ltd Quetiapine (seroquel). Product monograph 1998 Zeneca, London, UK 1 25

[B18] Ostapowicz G Fontana RJ Schiødt FV Larson A Davern TJ Han SH McCashland TM Shakil AO Hay JE Hynan L Crippin JS Blei AT Samuel G Reisch J Lee WM US Acute Liver Failure Study Group Results of a prospective study of acute liver failure at 17 tertiary care centers in the United States Ann Intern Med 2002 137 947 954 12484709 10.7326/0003-4819-137-12-200212170-00007

[B19] Bénichou C Criteria of drug-induced liver disorders. Report of an international consensus meeting J Hepatol 1990 11 272 276 2254635 10.1016/0168-8278(90)90124-a

[B20] Larrey D Epidemiology and individual susceptibility to adverse drug reactions affecting the liver Semin Liver Dis 2002 22 145 155 10.1055/s-2002-30101 12016546

[B21] Danan G Benichou C Causality assessment of adverse reactions to drugs–I. A novel method based on the conclusions of international consensus meetings: application to drug-induced liver injuries J Clin Epidemiol 1993 46 1323 1330 10.1016/0895-4356(93)90101-6 8229110

[B22] O’Grady JG Alexander GJ Hayllar KM Williams R Early indicators of prognosis in fulminant hepatic failure Gastroenterology 1989 97 439 445 2490426 10.1016/0016-5085(89)90081-4

